# P-1447. T-Cell Responses Following Two Doses of mRNA-1345 in Adult Solid Organ Transplant Recipients

**DOI:** 10.1093/ofid/ofaf695.1633

**Published:** 2026-01-11

**Authors:** Erick F Mayer, Cameron R Wolfe, Ann R Falsey, Christina Grassi, Avi Collins, Md Hasan, Hsiaohsuan Kuo, Shannon McGrath, Archana Kapoor, Xiaolin Chang, Xing Chen, Lan Lan, Sonia K Stoszek, Eleanor Wilson, Jaya Gowami, Rituparna Das, Frances Priddy

**Affiliations:** Moderna, Inc., Cambridge, MA; Duke University, Durham, NC; University of Rochester School of Medicine, Rochester, New York; Moderna, Inc., Cambridge, MA; Moderna, Inc., Cambridge, MA; Moderna, Inc., Cambridge, MA; Moderna, Inc., Cambridge, MA; Moderna, Inc., Cambridge, MA; Moderna, Inc., Cambridge, MA; Moderna, Inc., Cambridge, MA; Moderna, Inc., Cambridge, MA; Moderna, Inc., Cambridge, MA; Moderna, Inc., Cambridge, MA; Moderna, Inc., Cambridge, MA; Moderna, Inc., Cambridge, MA; Moderna, Inc., Cambridge, MA; Moderna, Inc., Cambridge, MA

## Abstract

**Background:**

Solid organ transplant recipients (SOTRs) are at elevated risk for severe respiratory syncytial virus (RSV) disease due to chronic immunosuppression. Cell-mediated immune responses are likely an important component of long-lasting protection against RSV. This substudy evaluates RSV-F–specific T-cell responses following 1 and 2 doses of mRNA-1345 50 µg in adult SOTRs.

**Figure 1.**
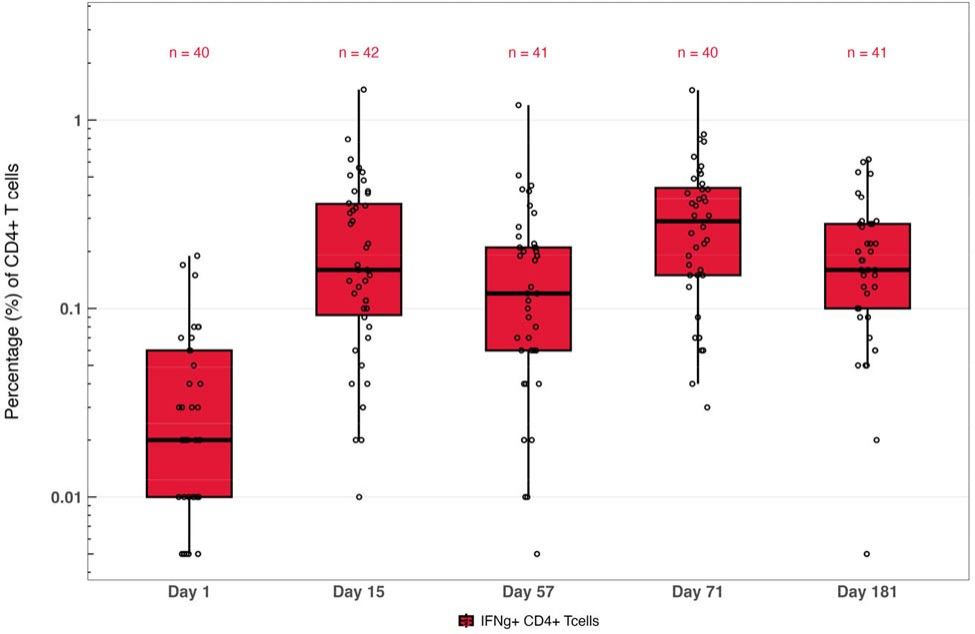
RSV PreF-Specific CD4+ Th1 Responses (IFNγ+) Through 6 Months After Vaccination With mRNA-1345 (50 µg) on Day 1 and Day 57 in Adult SOTRs

**Methods:**

Adults ≥18 years with a kidney, liver, or lung transplant received 2 doses of mRNA-1345 50 µg administered 57 days apart in an ongoing, phase 3 trial (NCT06067230). Peripheral blood mononuclear cells were optionally collected from a subset of participants at baseline (Day 1, pre-dose 1), Day 15, Day 57 (pre-dose 2), Day 71, and Day 181. RSV-F (prefusion conformation) T-cell responses were assessed; CD4+ and CD8+ cells were evaluated with intracellular cytokine staining for Th1 (IFN, TNF, IL2) or Th2 (IL4, IL5, IL13) responses, in addition to other functional markers (ie, CD40L, a marker of B cell co-stimulation).

**Results:**

Among 42 SOTRs (24 kidney, 8 lung, 8 liver, and 2 combined liver-kidney), RSV-F–specific CD4+ Th1 responses increased following the first mRNA-1345 dose, with an additional increase observed after Dose 2. Responses remained elevated through Day 181 (Figure). Polyfunctional CD4+ Th1 cells (expressing ≥2 or ≥3 cytokines) followed a similar trajectory. CD8+ Th1 responses were observed by Day 57. CD4+ Th2 responses showed minimal increases after Dose 1 and Dose 2, with no evidence of polyfunctionality, and were trending downward by Day 181. These trends were observed consistently across transplant types.

**Conclusion:**

mRNA-1345 50 µg elicited Th1-biased CD4+ T-cell responses with minimal and transient Th2 activation in adult SOTRs. CD8+ T-cell responses were also observed. T-cell responses persisted for 6 months post-vaccination. Findings demonstrate the ability of mRNA-1345 to induce cellular immunity in severely immunocompromised population, with durable responses which may be important for prevention of severe disease over time.

**Disclosures:**

Erick F. Mayer, MD, Moderna, Inc.: Employee of Moderna, Inc.|Moderna, Inc.: Stocks/Bonds (Public Company) Ann R. Falsey, MD, ADMA Biologics: Advisor/Consultant|ADMA Biologics: Honoraria|AstraZeneca: Advisor/Consultant|AstraZeneca: Grant/Research Support|AstraZeneca: Honoraria|CynaVac: Grant/Research Support|GSK: Advisor/Consultant|GSK: Honoraria|Merck: Advisor/Consultant|Merck: Honoraria|Moderna, Inc.: Advisor/Consultant|Moderna, Inc.: Grant/Research Support|Moderna, Inc.: Honoraria|Pfizer: Grant/Research Support|Sanofi Pasteur: Advisor/Consultant|Sanofi Pasteur: Honoraria Christina Grassi, MD, Moderna, Inc.: Employee of Moderna, Inc.|Moderna, Inc.: Stocks/Bonds (Public Company) Avi Collins, BScN, Moderna, Inc.: Former Moderna, Inc. Employee|Moderna, Inc.: Stocks/Bonds (Public Company) Md Hasan, PhD, Moderna, Inc.: Employee|Moderna, Inc.: Stocks/Bonds (Public Company) Hsiaohsuan Kuo, PhD, Moderna, Inc.: Employee|Moderna, Inc.: Stocks/Bonds (Public Company) Shannon McGrath, MS, Moderna, Inc.: Employee|Moderna, Inc.: Stocks/Bonds (Public Company) Archana Kapoor, PhD, Moderna, Inc.: Employee of Moderna, Inc.|Moderna, Inc.: Stocks/Bonds (Public Company) Xiaolin Chang, PhD, Moderna, Inc.: Moderna, Inc. employee|Moderna, Inc.: Stocks/Bonds (Public Company) Xing Chen, Sc.D., Moderna, Inc.: Moderna, Inc. employee|Moderna, Inc.: Stocks/Bonds (Public Company) Lan Lan, PhD, Moderna, Inc.: Employee of Moderna, Inc.|Moderna, Inc.: Stocks/Bonds (Public Company) Sonia K. Stoszek, PhD, Moderna, Inc.: Employee of Moderna, Inc.|Moderna, Inc.: Stocks/Bonds (Public Company) Eleanor Wilson, MD, MHS, Moderna, Inc.: Employee of Moderna, Inc.|Moderna, Inc.: Stocks/Bonds (Public Company) Jaya Gowami, MD, Moderna, Inc.: Employee of Moderna, Inc.|Moderna, Inc.: Stocks/Bonds (Public Company) Rituparna Das, M.D., Moderna, Inc.: Employee|Moderna, Inc.: Stocks/Bonds (Public Company) Frances Priddy, MD, MPH, Moderna, Inc.: Employee of Moderna, Inc.|Moderna, Inc.: Stocks/Bonds (Private Company)

